# Supplementary material for: The Photoconvertible Fluorescent Probe, CaMPARI, Labels Active Neurons in Freely-Moving Intact Adult Fruit Flies
Source: Front Neural Circuits. 2020 May 8;14:22. doi: 10.3389/fncir.2020.00022 (PMC7227398; doi:10.3389/fncir.2020.00022)
Supplement: Supplementary file 1 [file Data_Sheet_1.docx]

***Supplementary Material***


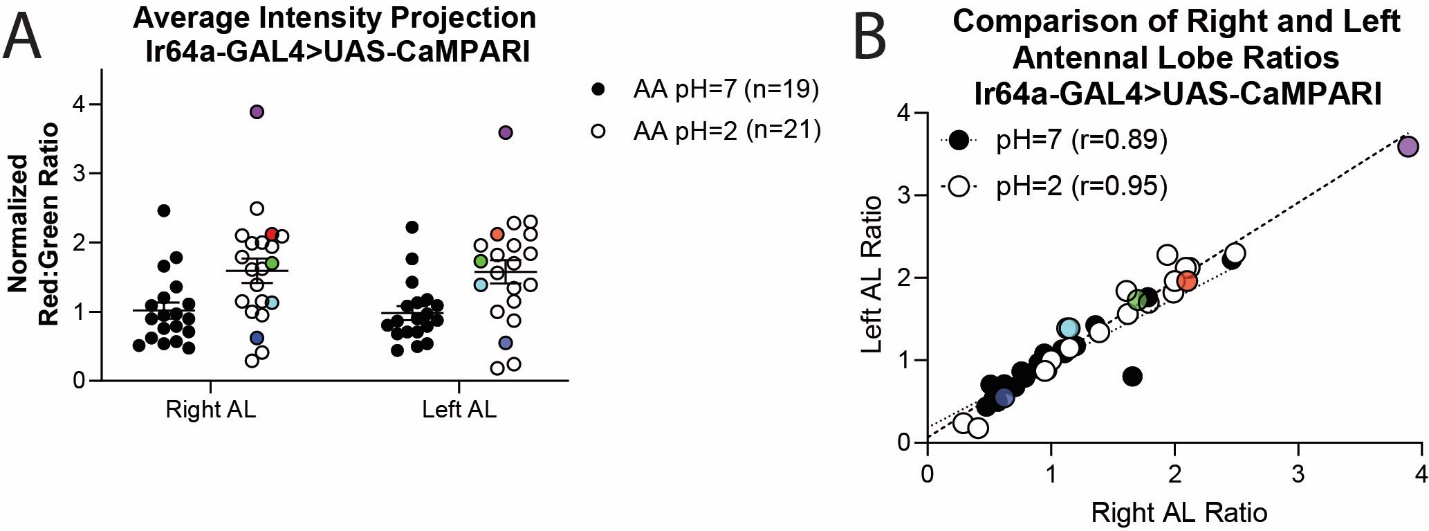
**1 Supplementary Figures**

**Figure S1: CaMPARI Photoconversion Is Comparable Between Right and Left Glomeruli Measured from the Same Brain**

**(A)** The Red:Green ratio of flies exposed to AA of pH=2 and pH=7 for 30 minutes. Individual measurements are shown for each right and left antennal lobe (AL) of an adult fly brain average intensity projection. Five color-matched paired points demonstrate that measurements taken from both glomeruli of the same brain are similar and so have been averaged together in subsequent graphs to give one point for each fly brain.**(B)** A direct comparison of the Red:Green ratios for each right and left AL demonstrates that measurements taken from the same brain are comparable. Right and left AL are correlated with a slope of 0.78 and r=0.89 for flies exposed to neutralized acetic acid and a slope of 0.95 and r=0.95 for flies exposed to acetic acid (pH=2).


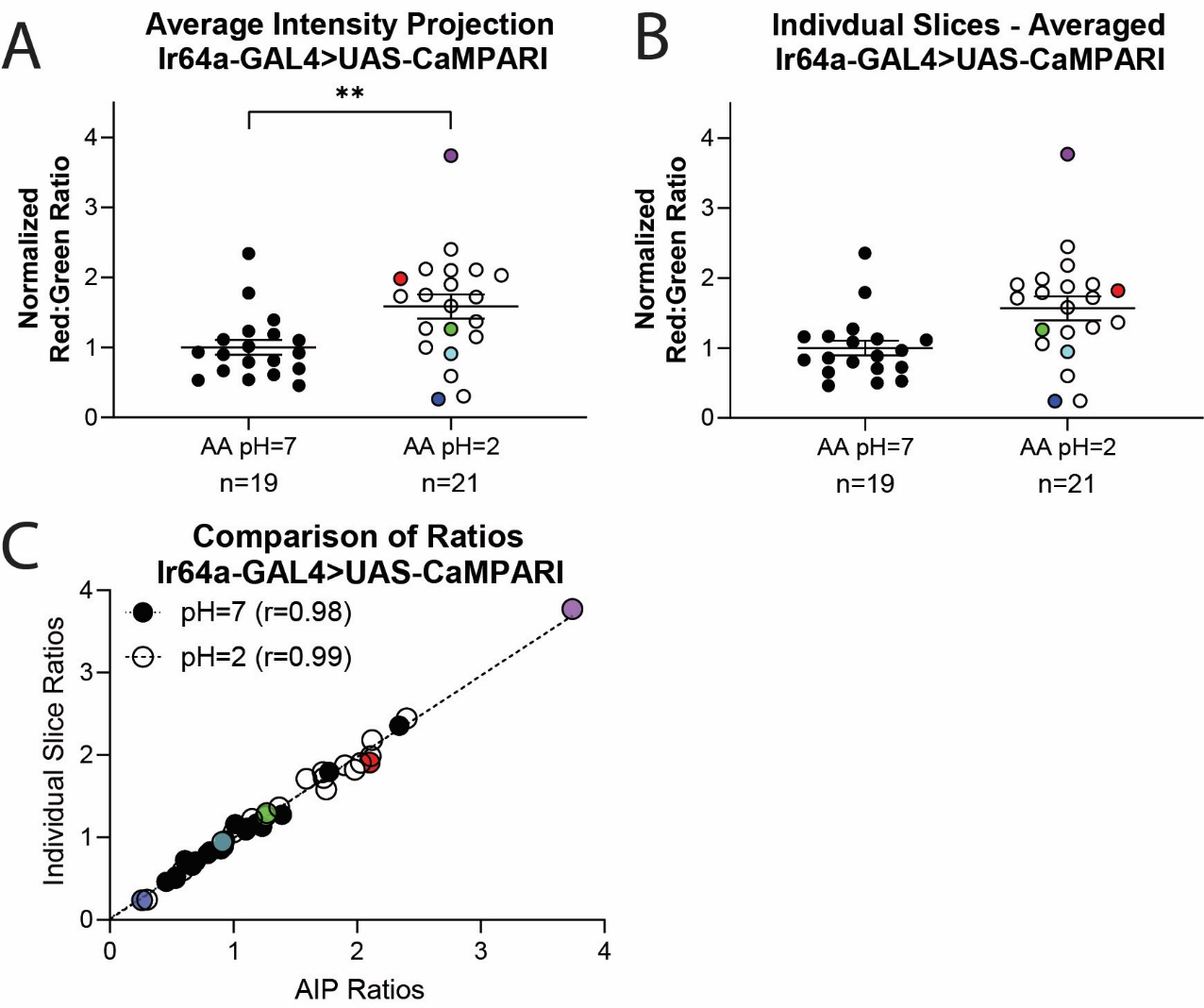


**Figure S2: Photoconversion Ratios Measured from Average Intensity Projection Images are Similar to Measurements from Individual Slices Averaged Together**

**(A-B)** The Red:Green ratio of flies exposed to AA of pH=2 and pH=7 for 30 minutes from adult fly brain average intensity projection (AIP) images **(A)** or individual slices containing the region of interest averaged together **(B)**. Five color-matched paired points demonstrate that measurements taken from AIP images are similar to measurements taken from images of the same fly brain where individual slice measurements have been averaged together. All subsequent graphs show measurements taken from AIP images. **(C)** A direct comparison of the Red:Green ratios for AIP measurements and individual slice measurements demonstrating measurements taken from the same brain are comparable. Right and left AL are correlated with a slope of 0.98 and r=0.98 for flies exposed to neutralized acetic acid and a slope of 0.98 and r=0.99 for flies exposed to acetic acid (pH=2).


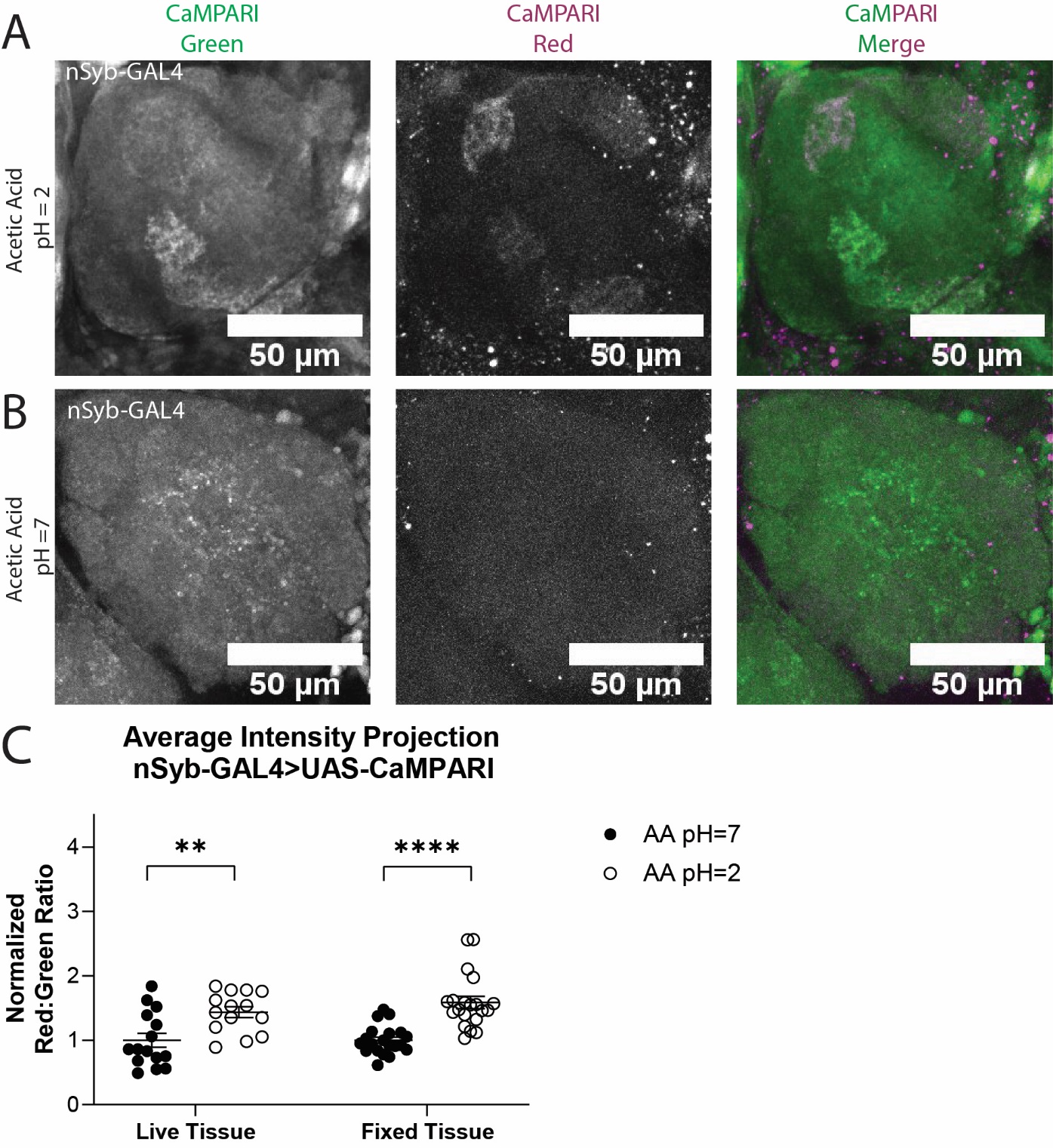


**Figure S3:** **Photoconversion Ratios Quantified from Live Tissue are Comparable to those from Fixed Tissue**

**(A-B)** Representative maximum intensity projection (MIP) images of adult fly antennal lobe expressing CaMPARI in nSyb-expressing neurons exposed to 5% acetic acid (AA) at pH=2 **(A)** or pH=7 **(B)** for 30 minutes. Photoconversion is present in Ir64a-expressing neurons in **(A)** when the pH=2 however no appreciable photoconversion is seen in **(B)** when the pH=7. **(C)** The Red:Green ratio of flies exposed to AA of pH=2 and pH=7 for 30 minutes for live and fixed tissue. Flies exposed to AA pH=2 (n=14) and imaged with live tissue have 1.4-fold greater photoconversion compared to flies exposed to AA pH=7 (n=15). **P=0.0056, Mann-Whitney U-test. In comparison, flies exposed to AA pH=2 and imaged with fixed tissue (n=20) have 1.6-fold greater photoconversion compared to flies exposed to AA pH=7 (n=20). ****P<0.0001, Mann-Whitney U-test.


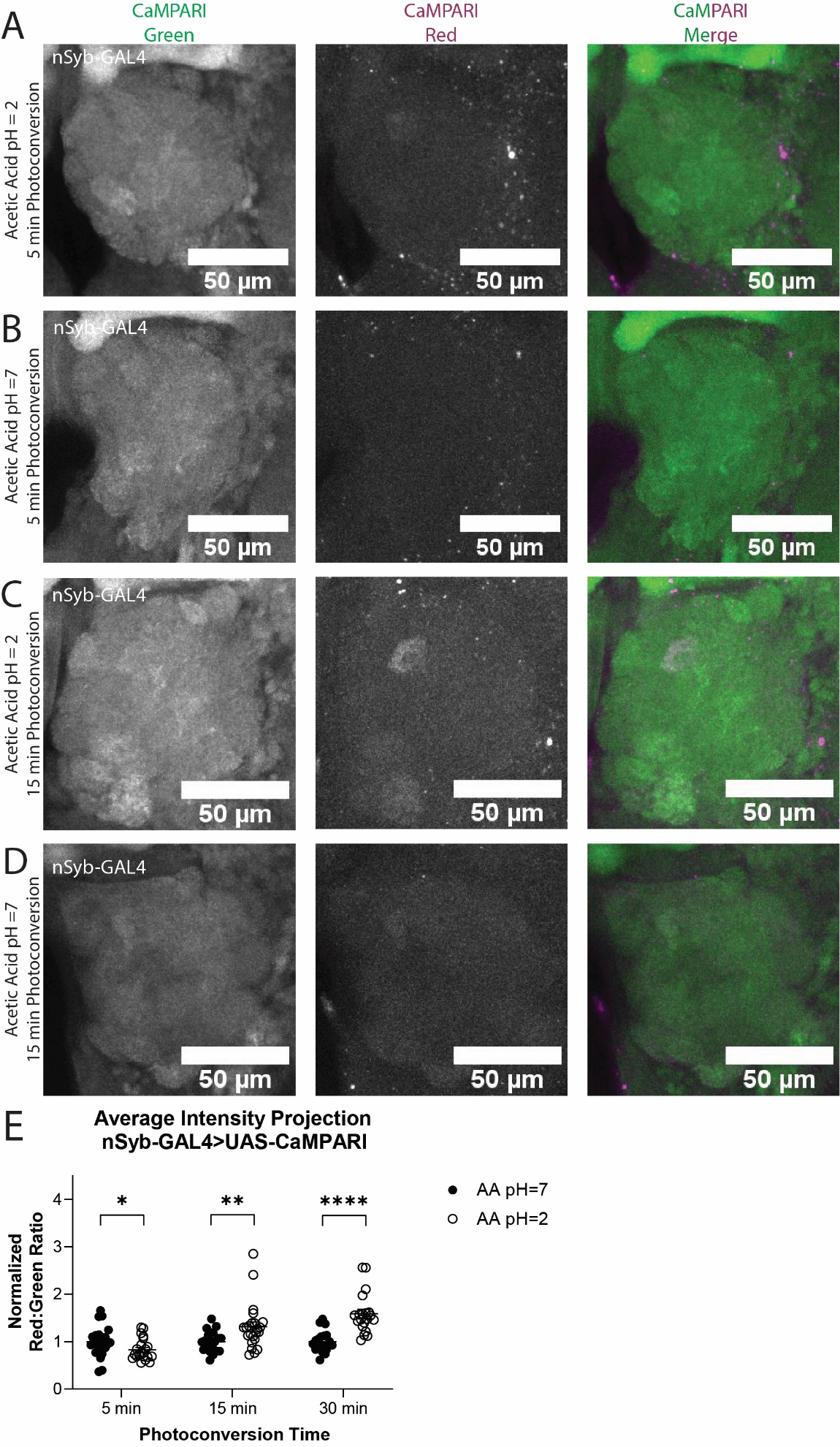


**Figure S4:** **Photoconversion Ratios of CaMPARI at 30 minutes is Higher than Earlier Time Points**

**(A-B)** Representative maximum intensity projection (MIP) images of adult fly antennal lobe expressing CaMPARI in nSyb-expressing neurons exposed to 5% acetic acid (AA) at pH=2 **(A)** or pH=7 **(B)** for 5 minutes. **(C-D)** Representative maximum intensity projection (MIP) images of adult fly antennal lobe expressing CaMPARI in nSyb-expressing neurons exposed to 5% acetic acid (AA) at pH=2 **(C)** or pH=7 **(D)** for 15 minutes. **(E)** The Red:Green ratio of flies exposed to AA of pH=2 and pH=7 for 5, 15 or 30 minutes. Flies exposed to AA pH=2 (n=23) for 5 mins have 0.8-fold photoconversion compared to flies exposed to AA pH=7 (n=22). *P=0.0217, Mann-Whitney U-test. For 15 mins, flies exposed to AA pH=2 (n=23) had 1.3-fold greater photoconversion compared to flies exposed to AA pH=7 (n=20). **P=0.0068, Mann-Whitney U-test. In comparison, flies exposed to AA pH=2 for 30 mins (n=20) have 1.6-fold greater photoconversion compared to flies exposed to AA pH=7 (n=20). ****P<0.0001, Mann-Whitney U-test.
